# Influence of patient characteristics on preventive service delivery and general practitioners’ preventive performance indicators: A study in patients with hypertension or diabetes mellitus from Hungary

**DOI:** 10.1080/13814788.2018.1491545

**Published:** 2018-08-02

**Authors:** János Sándor, Attila Nagy, Tibor Jenei, Anett Földvári, Edit Szabó, Orsolya Csenteri, Ferenc Vincze, Valéria Sipos, Nóra Kovács, Anita Pálinkás, Magor Papp, Gergely Fürjes, Róza Ádány

**Affiliations:** a Department of Preventive Medicine, Faculty of Public Health, University of Debrecen, Debrecen, Hungary;; b National Institute on Health Development, Department of Primary Health Care, Budapest, Hungary;; c WHO Collaborating Centre on Vulnerability and Health, Department of Preventive Medicine, Faculty of Public Health, University of Debrecen, Debrecen, Hungary;; d MTA-DE-Public Health Research Group, University of Debrecen, Debrecen, Hungary

**Keywords:** Guideline adherence, preventive services use, monitoring, primary care, risk adjustment

## Abstract

**Background:** Regular primary healthcare (PHC) performance monitoring to produce a set of performance indicators for provider effectiveness is a fundamental method for improving guideline adherence but there are potential negative impacts of the inadequate application of this approach. Since performance indicators can reflect patient characteristics and working environments, as well as PHC team contributions, inadequate monitoring practices can reduce their effectiveness in the prevention of cardiometabolic disorders.

**Objectives:** To describe the influence of patients’ characteristics on performance indicators of PHC preventive practices in patients with hypertension or diabetes mellitus.

**Methods:** This cross-sectional analysis was based on a network of 165 collaborating GPs. A random sample of 4320 adults was selected from GP’s patient lists. The response rate was 97.3% in this survey. Sociodemographic status, lifestyle, health attitudes and the use of recommended preventive PHC services were surveyed by questionnaire. The relationship between the use of preventive services and patient characteristics were analysed using hierarchical regression models in a subsample of 1659 survey participants with a known diagnosis of hypertension or diabetes mellitus.

**Results:** Rates of PHC service utilization varied from 18.0% to 97.9%, and less than half (median: 44.4%; IQR: 30.8–62.5) of necessary services were used by patients. Patient attitude was as strong of an influencing factor as demographic properties but was remarkably weaker than patient socioeconomic status.

**Conclusion:** These findings emphasize that PHC performance indicators have to be evaluated concerning patient characteristics.

KEY MESSAGES

- In Hungarian primary care, less than half of necessary preventive services for adult patients with hypertension or diabetes mellitus are delivered.
- Patients’ sociodemographic characteristics and health attitude significantly affect the utilization rates.
- Interventions for improving utilization rates have to be based on general practice level performance indicators adjusted for patients’ characteristics.

## Introduction

The exploitation of primary healthcare (PHC) opportunities is essential in tackling the most critical public health problems beyond just extending the range of services available at the PHC level [1]. This requires improving the quality of services provided, which depends on high rates of guideline adherence. It is widely accepted that the application of PHC performance monitoring is a fundamental method for achieving high adherence to guidelines since this approach can be used to assess the relative effectiveness and to make regular comparative evaluations of PHC practices [2,3]. This assertion has been demonstrated by the PHC performance monitoring systems that operate in many countries [4–7].

Although the usefulness of general practitioner (GP) performance monitoring is apparent, there are well-known problems with this approach [8–13]. These potential negative impacts and difficulties with implementation emphasize that only reliable provider indicators are useful in managing PHC problems and highlights the necessity of refraining from monitoring if the set of indicators is not complete.

Based on PHC monitoring experience, only a small proportion of the variability in provider indicators can be explained by the quality of the PHC team’s professional contribution. The main determining factors are patient characteristics and the parameters of the institutional environment [14–17]. Therefore, systems that utilize monitoring results should primarily consider essential provider indicators as a reflection of the critical working environment (where an above-average professional effort can achieve target performance because of a working environment of above-average difficulty). Accepting that both disadvantageous patient characteristics and an unfavourable institutional environment can be the base cause of difficulties, the explanation for the critical indicator value is rarely a low level of professional contribution by the PHC team [18,19].

Consequently, the preparation of an intervention based on an alarming indicator value requires an understanding of the causes of low performance.

If the main contribution of monitoring is not the identification of outlier providers but rather the understanding of the causes of performance variability, then there is a need to extend monitoring by incorporating as many patient characteristics as possible. This task is more difficult than using data related to the processes and outcomes that are registered somewhere in the usual health documentation of patients.

Our study on patients with hypertension or diabetes mellitus aimed to describe the influence of the patient characteristics (sociodemographics, lifestyles, and health attitudes) on performance indicators for recommended PHC preventive services and to demonstrate the constraints of using performance indicators without adjusting for patient characteristics.

## Methods

### Study design

This study used a questionnaire-based survey involving a network of 165 GPs, who were organized for participation in a primary care model-programme in Hungary. The programme aimed to develop or improve the preventive services available at the PHC level [20].

### Setting and study population

The network consists of a group of GPs involved in developing new services and another group providing reference data to evaluate the effectiveness of these efforts. Data analysed in the present study came from a random sample of adults collected using a baseline survey before service development.

The ‘new services developing group’ consisted of 34 GPs. A list of adults aged 18 or older compiled by these GPs was used as a sampling frame. Fifty adults from each practice were randomly selected, resulting in a sample of 1700 people. The General Practitioners Morbidity Monitoring System (GPMMS) [21] organized the ‘reference data producing group’. The sampling frame of adults aged 18 or older and registered within one of the 131 GPMMS practices was used to select randomly 20 subjects from each practice. This sample consisted of 2620 people.

**Figure 1.**
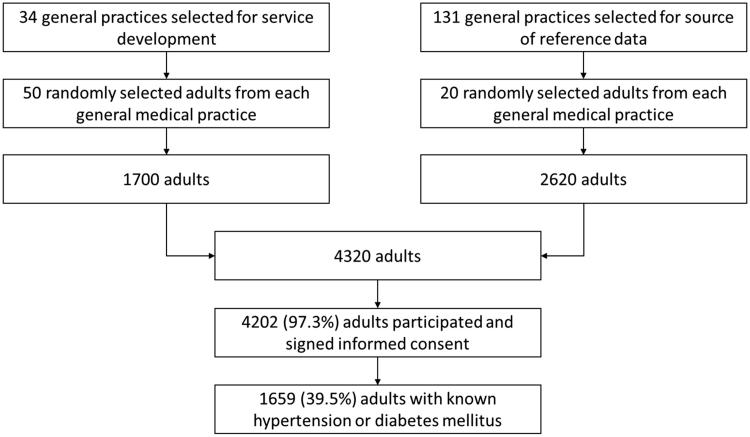
Selection of study patients from participants of the baseline survey of a primary healthcare service development programme.

Thus, 4320 subjects were selected for the study; of these, 4202 signed the informed consent (97.3%). Adults with hypertension or diabetes mellitus known by GP irrespective of their treatment status were identified in the two parts of the sample, and the data obtained for these patients were analysed in the present study (1659 patients; see Figure 1).

### Data collection

The questionnaire surveyed the cardiometabolic risk status and use of preventive PHC services by subjects. The nurses employed in the participating medical practices were the interviewers. They visited personally (many times if it was needed) the selected subjects if they did not respond to the invitation. The data collection started in December 2012 and finished in July 2013.

### Variables

#### Assessment of sociodemographic characteristics and lifestyle variables

Questions were taken from the Hungarian implementation of the first wave of the European Health Interview Survey [22].

- Sociodemographic factors: age (in 15–44, 45–64, 65–X year groups), sex, education level (primary or less, vocational, high school, and tertiary), ethnicity (the only ethnic minority in Hungary is Roma, who have a disadvantageous health status in general; self-declaration classified a Roma ethnicity), and eligibility for a prescription exemption certificate (which ensures free of charge access to medicines and medical devices for patients living in deprivation).
- Lifestyle: smoking behaviour (regular, every day smoker or not), alcohol misuse (CAGE classification), body mass index (underweight < 20; 20 ≤ normal <25, 25 ≤ overweight < 30, obese ≥ 30; patient-reported data), and central obesity (measuring waist circumference; healthy thresholds: men ≤ 94, women ≤ 80).

#### Health attitudes

Assessed using eight questions (see Table 1) from the Health Education Monitoring Survey (UK) of the Office for National Statistics [23], which had been used in a nationwide representative survey for WHO European Regional Office to describe the health attitude of Hungarian adults (unpublished report). Respondents were asked to rate their answers on a scale of agreement (strongly agree, agree, neither agree nor disagree, disagree, strongly disagree). The frequency of strong agreement was calculated for all statements.

**Table 1.** Proportion of adult patients with hypertension or diabetes mellitus, who strongly agreed with statements related to health attitudes, and the factors identified in the principal component analysis (*n* = 1659).

Statements/health attitudes	Proportion of strong agreement		Factor loading		
	%	95%CI	Factor 1	Factor 2	Factor 3
Responsibility-averting:					
I have to be very ill before I go to the doctor	34.0	31.7–36.3	0.767	0.044	0.109
If you overthink about your health, you are more likely to be ill	30.0	27.8–32.2	0.575	0.151	0.298
The most important thing is the constitution you are born with	29.4	27.2–31.6	0.528	0.223	0.319
People like me don’t really have time to think about their health	14.8	13.1–16.5	0.671	−0.114	0,061
Dedicated-to-health:					
To have good health is the most important thing in life	90.2	88.7–91.6	0.104	0.810	0.000
It’s sensible to do exactly what the doctors say	81.4	79.6–83.3	0.000	0.818	0.115
Faithful:					
Generally, health is a matter of luck	14.2	12.5–15.8	0.241	0.091	0.759
Suffering sometimes has a divine purpose	12.2	10.6–13.8	0.147	0.014	0.776

#### Use of preventive services (as prescribed for patients with a cardiometabolic disease by Decree no. 51/1997 of the Minister of Welfare)

Investigated by collecting the following variables: measurement of blood pressure, serum glucose, serum lipid parameters, urinary creatinine and urinary protein (in 12 months); assessment (in 24 months) of family history, dietary habit, smoking habit; measurement (in 24 months) of body weight and waist circumference); atherosclerosis evaluation over 40 years of age (in 24 months); screening (in 24 months) for alcohol misuse and oral cavity disorders; visual acuity and hearing loss over 65 years of age (in 36 months); participation (motivated by GPs) in screening for breast cancer (women 45–65 years, in 24 months), cervical cancer (women 25–65 years, in 36 months), prostate cancer (men 65+ years, in 36 months), and colorectal cancer (50–75 years, in 36 months).

#### Performance indicators

The counselling by GP (CGP) and physical examination by GP (PGP) indicators summarize family history, alcohol use, smoking and nutrition-related counselling activities of GPs, and body weight, waist circumference, blood pressure, oral cavity status, vision loss and hearing loss examinations by GPs, respectively. CGP and PGP indicators were aggregated into a counselling and physical examination by GP (CPGP) indicator. Referral to laboratory investigations (LAB) and referral to organized cancer screening (SCR) indicators combine serum glucose, serum lipid, urinary creatinine, and urinary protein measurements and cervical, breast, prostate, and colorectal cancer screening, respectively. The referral to secondary care (SEC) indicator summarizes the LAB and SCR indicators. The use of preventive services (UPS) indicator covers all of the preventive activities.

### Statistical analysis

The rate of PHC service utilization (proportion of adults who used preventive services) was calculated for each service, along with 95% confidence intervals 95%CIs). Summary measures, aggregated ratios (CGP, PGP, CPGP, LAB, SCR, SEC and UPS) were computed as the number of services utilized and the number of services that should have been used considering the age and sex of the patient. Medians with their corresponding interquartile ranges were calculated for these aggregated ratios.

Attitude-related variables were processed using principal component analysis with PROMAX rotation to reduce the number of explanatory variables introduced into the statistical models. The relationships between the use of preventive services and patient characteristics were analysed using hierarchical multivariate logistic regression models for each summary indicator of service use with general medical practice effects, separately. Median values of patients’ service use in the whole sample were applied to dichotomize the use of recommended preventive service outcome indicators to make a distinction between patients with higher than median and not higher than median service use. The dichotomized indicators were used as outcome variables in the regression models. The results are presented as odds ratios with 95%CIs. Analyses were performed using PASW Statistics version 18 (Armonk, New York, United States).

## Results

The participants’ characteristics are summarized in Table 2.

**Table 2.** Distribution of patients’ characteristics in the study sample (1659 adult patients with hypertension or diabetes mellitus).

Patient characteristic	Category	*n* (%)
Sex	Men	701 (42.3)
	Women	958 (57.7)
Age groups	15–44	189 (11.4)
	45–64	753 (45.4)
	65–X	717 (43.2)
Level of education	Primary or less	640 (38.6)
	Vocational	410 (24.7)
	High school	419 (25.3)
	Tertiary	163 (9.8)
	Not declared	27 (1.6)
Roma ethnicity		73 (4.4)
Eligible for prescription exemption certificate*		195 (11.8)
Regular (every day) smoker		303 (18.3)
Alcohol misuse (CAGE score ≥ 2)		158 (9.5)
BMI	Underweight (BMI < 20)	18 (1.1)
	Normal (20 ≤ BMI < 25)	340 (20.5)
	Overweight (25 ≤ BMI < 30)	600 (36.2)
	Obese (BMI ≥ 30)	701 (42.3)
Healthy waist circumference (men ≤ 94cm, women ≤ 80 cm)		1464 (88.2)

BMI: body mass index.

*Free of charge access to medicines and medical devices for patients living in deprivation.

### Health attitudes of patients

The distribution of opinions regarding health attitude statements is summarized in Table 1. The opinions were useful for factor analysis (Kaiser–Meyer–Olkin measure (KMO) 0.645); Bartlett’s test *P* < 0.001). Three factors had an eigenvalue greater than 1 and were responsible for 54.03% of the total variance. This analysis yielded three factors. Statements with a loading value higher than 0.5 were considered as main factor components. Factors 1, 2, and 3 were primarily determined by the 3rd, 4th, 5th, and 6th; the 1st, and 2nd; the 7th, and 8th statements, respectively. After evaluating the contents of factor-building statements, factors 1, 2, and 3 were referred to as responsibility averting, dedicated to health, and faithful attitudes, respectively. The resulting factor scores with a standard normal distribution were introduced into regression models as continuous explanatory variables.

### Preventive service use and performance indicators

Service use varied considerably (median: 38.6%; IQR: 30.63–62.1), from 18.0% for colorectal cancer screening to 97.9% for hypertension screening (Table 3).

**Table 3.** Unadjusted service utilization frequencies for the investigated preventive services among adult patients with hypertension or diabetes mellitus (*n* = 1659).

Preventive services	Service utilization frequencies	
	%	95%CI
Measurement of blood pressure in 12 months	97.9	97.2–98.6
Measurement of serum glucose in 12 months	80.2	78.3–82.2
Measurement of serum lipid parameters in 12 months	73.2	71.0–75.3
Measurement of body weight in 24 months	63.0	60.7–65.3
Cervical cancer screening (women, 25–65 years) in 36 months	62.7	58.4–67.1
Measurement of urinary proteins in 12 months	61.5	59.1–63.8
Breast cancer screening (women, 45–65 years) in 24 months	60.8	56.0–65.6
Measurement of urinary creatinine in 12 months	48.5	46.1–50.9
Assessment of family history in 24 months	40.3	38.0–42.7
Prostate cancer screening (men, 65+ years) in 36 months	38.6	32.4–44.8
Test of visual acuity in 36 months	36.2	33.9–38.5
Assessment of dietary habits in 24 months	33.5	31.2–35.8
Measurement of waist circumference in 24 months	32.9	30.7–35.2
Examination for atherosclerosis in 24 months	31.7	29.5–34.0
Screening for alcohol misuse in 24 months	29.5	27.3–31.7
Assessment of smoking habits in 24 months	24.7	22.6–26.8
Oral cavity cancer screening in 24 months	21.5	19.5–23.4
Test for hearing loss in 36 months	18.0	16.2–19.9
Colorectal cancer screening (50–75 years) in 36 months	18.0	15.6–20.4

The medians of summary performance indicators varied between 0% (the median subject was not referred to organized cancer screening by the GP at all) and 75% (the median subject was referred to 75% of necessary laboratory investigations by the GP) (Table 4).

**Table 4.** Preventive service utilization in the studied sample of adult patients with hypertension or diabetes mellitus, and the median ratio^a^ of patients’ preventive service utilization within groups of preventive services (*n* = 1659).

	In the sample			
Groups of preventive services	Number of implemented interventions	Number of required interventions	Service utilization frequency	Median (interquartile range) ratio of service utilization
Single preventive services				
Referral to laboratory investigations^b^	4369	6636	65.8%	75% (25–100)
Physical examination by GP^c^	4889	9612	50.9%	42.9% (28.6–57.1)
Referral to organized cancer screening^d^	812	2116	38.4%	0% (0–66.7)
Counselling by GP^e^	2125	6636	32.0%	25% (0–50)
Combined preventive services				
GP services: counselling and physical examination^b^^+^^d^	7014	16248	43.2%	36.4% (18.2–54.6)
Secondary care services^b^^+^^d^	5181	8752	59.2%	66.7% (40–83.3)
Overall use of preventive services^b^^+^^c^^+^^d^^+^^e^	12195	25000	48.8%	44.4% (30.8–62.5)

a As the number of utilized to the number of recommended preventive services.

b Serum glucose, serum lipid, urinary creatinine, and urinary protein measurements.

c Body weight, waist circumference, blood pressure, oral cavity status, vision loss and hearing loss examinations.

d Cervical, breast, prostate, and colorectal cancer screening.

e Family history, alcohol use, smoking and nutrition-related counselling.

### Patient factors influencing preventive service use

The nature of the associations between the patient characteristics and service utilization rates are shown in Table 5.

**Table 5.** Patient characteristics influencing the utilization of preventive services by GPs according to hierarchical multivariate logistic regression models with general practice effects (ORs with 95%CIs; *n* = 1659).

	Counselling by GP^a^	Physical examination by GP^b^	GP services: counselling and physical examination^a^^+^^b^	Referral to laboratory investigations^c^	Referral to organized cancer screening^d^	Secondary care services^c^^+^^d^	Overall use of preventive services^a^^+^^b^^+^^c^^+^^d^
Sociodemographic factors							
Sex (women/men)	**0.67(0.57–0.79)**	**0.81(0.70–0.94)**	**0.69(0.60–0.81)**	0.97 (0.84–1.12)	**4.38(3.68–5.22)**	**1.30(1.14–1.50)**	**0.83(0.72–0.95)**
Age (<45/65+)	1.22 (0.96–1.56)	**0.68(0.54–0.85)**	**0.78(0.62–0.99)**	**0.74(0.59–0.93)**	**4.53(3.17–6.47)**	**1.57(1.26–1.95)**	1.08 (0.86–1.35)
Age (45–64/65+)	0.95 (0.81–1.13)	0.91 (0.78–1.06)	0.83 (0.71–0.97)	0.94 (0.81–1.09)	**2.67(2.26–3.17)**	1.08 (0.94–1.25)	**0.81(0.70–0.95)**
Education (vocational/primary or less)	1.04 (0.84–1.28)	**1.29(1.07–1.56)**	**1.25(1.03–1.51)**	**1.44(1.20–1.72)**	1.18 (0.96–1.46)	1.17 (0.98–1.38)	**1.34(1.12–1.6)**
Education (high school/primary or less)	**1.31(1.07–1.6)**	**1.82(1.52–2.18)**	**1.60(1.33–1.93)**	**1.90(1.59–2.28)**	**1.45(1.17–1.78)**	**1.51(1.27–1.79)**	**1.82(1.52–2.17)**
Education (tertiary/primary or less)	**1.75(1.35–2.28)**	**1.99(1.56–2.53)**	**1.88(1.47–2.41)**	**2.27(1.78–2.90)**	**1.88(1.43–2.48)**	**1.41(1.11–1.77)**	**2.25(1.76–2.87)**
Ethnicity (Roma/non-Roma)	**1.67(1.11–2.5)**	**1.43(1.01–2.00)**	**2.39(1.68–3.41)**	1.02 (0.74–1.42)	**2.06(1.37–3.08)**	1.08 (0.79–1.49)	1.24 (0.89–1.74)
Prescription exemption certificate (eligible/ineligible)	**1.72(1.38–2.15)**	**1.80(1.47–2.20)**	**1.80(1.46–2.21)**	**1.91(1.56–2.35)**	1.12 (0.88–1.42)	**1.70(1.4–2.07)**	**2.18(1.77–2.67)**
Lifestyle factors							
Regular smoking	**1.74(1.43–2.11)**	0.91 (0.76–1.09)	**1.22(1.01–1.46)**	**0.71(0.59–0.84)**	0.85 (0.69–1.04)	**0.73(0.62–0.87)**	**1.19(1.00–1.42)**
Alcohol misuse	**1.96(1.53–2.5)**	1.13 (0.90–1.42)	**1.52(1.21–1.92)**	0.84 (0.67–1.05)	**0.74(0.56–0.98)**	0.89 (0.72–1.10)	0.88 (0.70–1.1)
BMI (obese/normal)	1.18 (0.94–1.47)	1.02 (0.84–1.24)	1.02 (0.83–1.24)	**1.62(1.33–1.97)**	**0.64(0.51–0.81)**	**1.31(1.09–1.57**)	1.17 (0.97–1.42)
BMI (over-weigh/normal)	**1.28(1.03–1.59)**	0.87 (0.71–1.06)	0.98 (0.80–1.19)	**1.30(1.07–1.58)**	0.83 (0.66–1.04)	1.15 (0.96–1.37)	1.00 (0.83–1.21)
BMI (underweight/normal)	**2.36(1.09–5.12)**	0.65 (0.30–1.44)	0.53 (0.22–1.27)	0.87 (0.46–1.65)	**0.18(0.05–0.57)**	0.72 (0.40–1.33)	0.84 (0.42–1.65)
Central obesity	0.83 (0.64–1.08)	1.22 (0.95–1.55)	0.94 (0.73–1.20)	1.01 (0.80–1.27)	**1.41(1.06–1.88)**	1.09 (0.87–1.37)	1.04 (0.83–1.32)
Health attitudes							
Responsibility-averting	**0.90(0.83–0.97)**	**0.91(0.84–0.97)**	**0.81(0.75–0.87)**	**0.84(0.79–0.90)**	**0.84(0.77–0.91)**	**0.88(0.83–0.94)**	**0.80(0.74–0.85)**
Dedicated to health	**1.13(1.04–1.22)**	**1.08(1.01–1.17)**	**1.22(1.13–1.31)**	1.02 (0.95–1.09)	1.01 (0.92–1.11)	0.99 (0.93–1.06)	**1.21(1.13–1.3)**
Faithful attitude	**1.11(1.03–1.2)**	1.07 (0.99–1.14)	**1.13(1.05–1.21)**	**1.11(1.04–1.19)**	0.94 (0.87–1.03)	**1.10(1.03–1.18)**	**1.15(1.07–1.23)**

Bold: significant result.

a Family history, alcohol use, smoking and nutrition-related counselling.

b Body weight, waist circumference, blood pressure, oral cavity status, vision loss and hearing loss examinations.

c Cervical, breast, prostate, and colorectal cancer screening.

d Serum glucose, serum lipid, urinary creatinine, and urinary protein measurements.

OR: odds ratio; 95%CI: 95% confidence interval; BMI: body mass index.

#### Sociodemographic factors

The female sex was positively associated with SCR (and consequently with SEC) and inversely related to the direct actions of GP (CGP, PGP, CPGP). Older age was less frequently associated with CGP, PGP, CPGP, and SCR. LAB was strongly associated with older subjects. A higher level of education was a decisive factor for each indicator. The Roma ethnicity showed association with CGP (and consequently with CPGP) and SCR. The patients with eligibility for prescription exemption certificates were more likely to be provided with both CGP and PGP (and consequently with CPGP), as well as with LAB (and thus with SEC).

#### Lifestyle factors

The only indicator that had a severity-dependent association with BMI was LAB. SCR alone showed a positive association with central obesity. CGP was more strongly associated with smoking assessment, and SEC was less frequent among regular smokers. Alcohol misuse was a negative factor for SCR, but a positive one for CGP (and consequently for CPGP).

#### Health attitudes

The responsibility-averting attitude was a factor in reducing the rate of PHC service utilization rates for all indicators apart from the PGP. The dedicated to health (for CGP, CPGP) and the faithful attitude (for CGP, CPGP, LAB, and SEC) proved to be significant influencing factors for more intensive service usage.

In general, the UPS was negatively associated with female sex, an older age, and a responsibility-averting attitude and positively with higher education, eligibility for a prescription exemption certificate, and dedicated to health, and faithful attitudes.

## Discussion

### Main findings

Our investigation demonstrated that the observed 44.4% median use of PHC related preventive services varied significantly by the patients’ age, sex, education, deprivation (indicated by the eligibility for prescription exemption certificates), and attitude.

Although there were considerable differences among the rates at which the investigated services were utilized, most rates were quite low. The socio-economic status indicated by education and eligibility for prescription exemption certificates proved to be the strongest and most consequential factor influencing preventive service use.

Regarding the summary indicator of UPS, patients’ health attitudes had a significant influence, but this effect was not observable for each studied performance indicator. Altogether, attitude had an impact similar to that of sex and age (taking into account that the age range in the studied population was 90 years).

Our results demonstrate that a disadvantageous health behaviour (indicated by regular smoking, alcohol misuse, central obesity, and a high BMI in our investigation) does not have similar importance in the provision of preventive services relative to its importance in determining the long-term outcomes of their cardiometabolic disorders.

### Strengths and limitations

The studied sample was selected at random, and the response rate was high, which prevented the patient level selection bias.

GP participation was voluntary, and it is likely that participants represent the GPs who are more committed to the quality of care. Consequently, the observed rates at which services were provided in the present study may be overestimated. This bias only limits the external validity of our research but does not limit the internal validity.

Because nurses who were involved in the care of the survey participants collected data on service use, it is probably well registered. However, there may be a small number of investigations carried out by secondary care providers that remained hidden due to incomplete reporting to the GP or inaccurate recall by the patients. Altogether, there is likely a small probability of an absent registration or the miscoding of service use.

However, services were not strictly defined in the study beyond the standard definitions, and the reported services could vary by content and quality (especially in the case of checks for arteriosclerosis). Our study could not evaluate the association between quality of preventive services and patient characteristics.

The study model did not cover the PHC organizational environment. For example, availability of laboratories and diagnostic facilities at secondary care providers, support of municipalities for PHC providers) were not covered by the data collection. Since all the patients of a particular GP are exposed to the same organizational environment, the lack of the control for these availability factors cannot influence the observed association between patient characteristics and preventive service use.

Since the attitudes were not assessed in a detailed way, and the study design precluded the evaluation of causality, further investigations are needed to clarify the mechanisms by which the patients’ attitudes and the preventive service utilization are associated. Our observations do not suggest that the patients’ limited willingness to cooperate with GPs is the only cause of preventive service failure, and do not exclude that the GPs’ attitude towards patients with certain attitude results in variability in service provided. The distribution of responsibility between patients and GPs in the failure of service use needs additional research.

### Results in relation to existing literature

The weak adherence to recommendations for primary care level preventive services observed in our investigation is in concordance with the general weakness of the Hungarian PHC system [24], and it suggests that Hungarian PHC teams do not strictly follow guidelines, which may contribute to the tenth highest hypertension and hyperglycaemia related mortality of the world [25]. Furthermore, the less than recommended level utilization of preventive services among patients with hypertension or diabetes mellitus is similar to that observed among adults without hypertension or diabetes mellitus observed in Hungary [26].

Our study confirmed formerly published results from Spain [14], Canada [15], and the US [16] on the significant role of patient characteristics on PHC service utilization rates. Our observations are in line with the conclusion from a review that the non-patient related factors are responsible for not more than 20% of the variability of healthcare quality indicators [27]. Furthermore, the better quality of hypertension or diabetes mellitus care for male and more aged patients was observed not only in our study but also in the UK [28] and in the US [29].

### Implications

These findings emphasize that to be useful in assessing PHC services, performance monitoring (which needs to recognize performance deviations and to detect the causes of performance deviations) has to identify patient characteristics (high-risk groups) associated with low-quality preventive care. Its goal should be to determine target groups of intervention in addition to identifying problems in the provision of preventive services for patients with hypertension or diabetes mellitus.

## Conclusion

Our results demonstrate that those patient characteristics, such as sociodemographic properties and health attitude are associated with the utilization of PHC preventive services. It suggests that variability in how PHC providers’ performance in providing preventive services is significantly associated with patient characteristics.
